# Maxillary Cast Partial Denture and Mandibular Implant-Supported Metal-Ceramic Prosthesis With a Split Framework to Compensate for Mandibular Flexure: A Case Report

**DOI:** 10.7759/cureus.49071

**Published:** 2023-11-19

**Authors:** Ananya, Priya Rani, Tushar Sinha, Jayant Prakash

**Affiliations:** 1 Prosthodontics, Rajendra Institute of Medical Sciences, Ranchi, IND; 2 Dentistry, Sadar Hospital, Muzaffarpur, IND

**Keywords:** implant-supported fixed prosthesis, framework splitting, mandibular flexure, cast partial denture, dental implants

## Abstract

The goal of modern dentistry is to restore optimum oral health, function, and comfort for a patient. For an implant-supported fixed prosthesis, these goals cannot be met if the biomechanical factors governing the success of the prosthesis are overlooked. Mandibular flexure is one such factor that needs to be considered, especially when implants are being placed posterior to the interforaminal region. If not, it can lead to problems like increased stress, bone resorption, poor fit of the prosthesis, screw loosening, and patient discomfort. The use of a split-framework prosthesis is one of the measures that could be taken to decrease the stress, ensure a passive fit of the framework, and long-term maintenance of patient comfort and function. This case report describes the oral rehabilitation of a patient using a maxillary cast partial denture and mandibular split framework fixed prosthesis to compensate for mandibular flexure.

## Introduction

Dental implants have gained popularity over the past few decades for the rehabilitation of edentulous patients and patients with implant-supported prostheses observed marked improvement in oral health, function, and comfort than patients with conventional removable prostheses [1]. However, the recent evolutions in implant dentistry emphasize the use of new geometries and prosthetic designs based on restorative and biomechanical properties and the behavior of implant rehabilitation [2].

One such factor is median mandibular flexure. It is the deformation of the mandible portrayed by the property of the mandible to flex inward with a reduction in the width of the mandibular arch during jaw opening and protrusion [3]. Several authors have demonstrated this sagittal movement of the posterior segment of the mandible due to the contraction of the lateral pterygoid muscle, which causes flexure of the mandible around the mental symphysis [4-8].

The amplitude of the median mandibular flexure increases as the opening or protrusive movement increases and as it approaches the ramus. It also varies with bone volume and density, age of the patient, gonial angle, symphyseal bone height, etc. The amplitude has been measured as 800 µm in the first molar to as much as 1500 µm in the ramus area, while the movement of the natural tooth ranges from 28 to 108 µm [9]. Median mandibular flexure poses testing problems for implant-supported prostheses. It leads to elevated stress in implant prostheses and abutments, ill-fitting prostheses, distortion of impression, implant screw fracture or porcelain crown fracture, pain during function, loosening of cemented prostheses, and resorption of bone around the implant. Hence, for a better outcome and longevity of implant and dental prostheses, it is important to minimize median mandibular flexure [10-14]. Splitting the prosthesis framework between the mental foramen has been suggested as one of the ways to effectively reduce mandibular flexure [9-13]. This article describes full mouth rehabilitation using a maxillary cast partial denture and mandibular implant-supported fixed prosthesis with a split framework to compensate for mandibular flexure.

## Case presentation

Clinical report

A 55-year-old female patient reported the chief complaint of missing maxillary teeth and pain and mobility in the remaining mandibular teeth. A detailed case history was recorded, the intraoral examination was done, and a total extraction concerning the mandibular arch was planned owing to generalized periodontitis, followed by mandibular implant-supported metal-ceramic prosthesis and maxillary cast partial denture.

After the remaining mandibular teeth were extracted, four implants (Alpha Bio Tec., Petah Tikva, Israel) were placed in the posterior mandible, and two implants (Alpha Bio Tec.) in the interforaminal region. After four months of uneventful healing and radiographic evaluation, second-stage surgery was planned.

**Figure 1:**
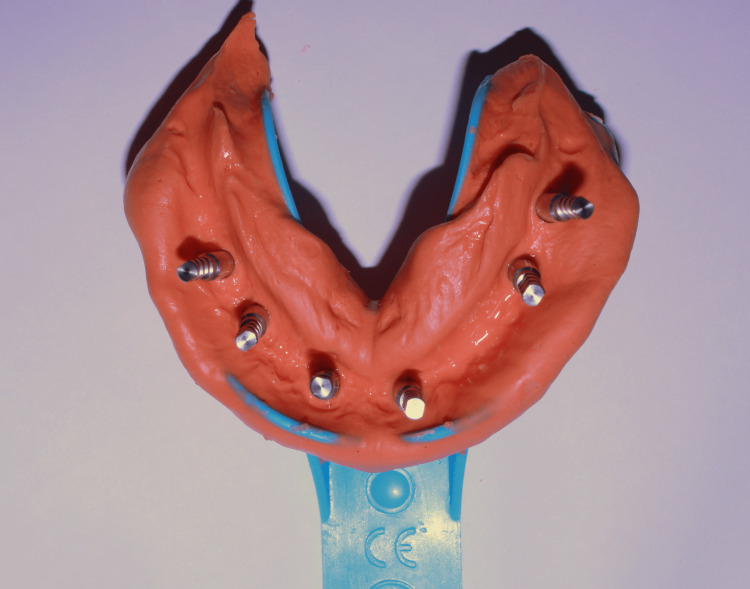
Alginate open tray impression

Punch incisions were made to expose the cover screws followed by their removal and placement of healing abutments. After two weeks, prosthetic rehabilitation was started. Open tray impression copings were placed in implant bodies and an alginate open tray impression was made (Figure 1) in alginate after tray customization to allow exposure of impression copings during impression making.

**Figure 2:**
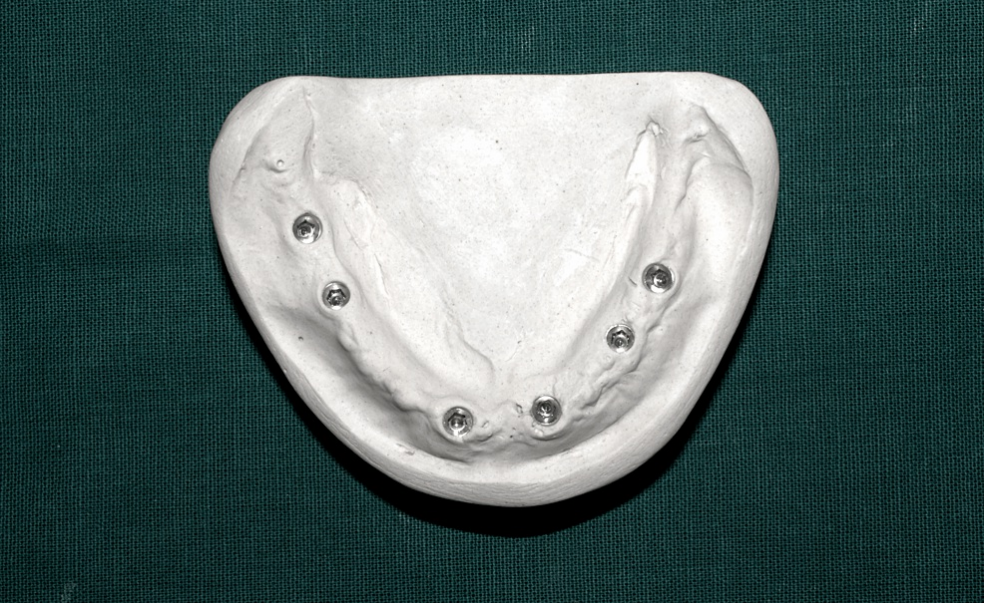
Primary cast poured in dental plaster

**Figure 3:**
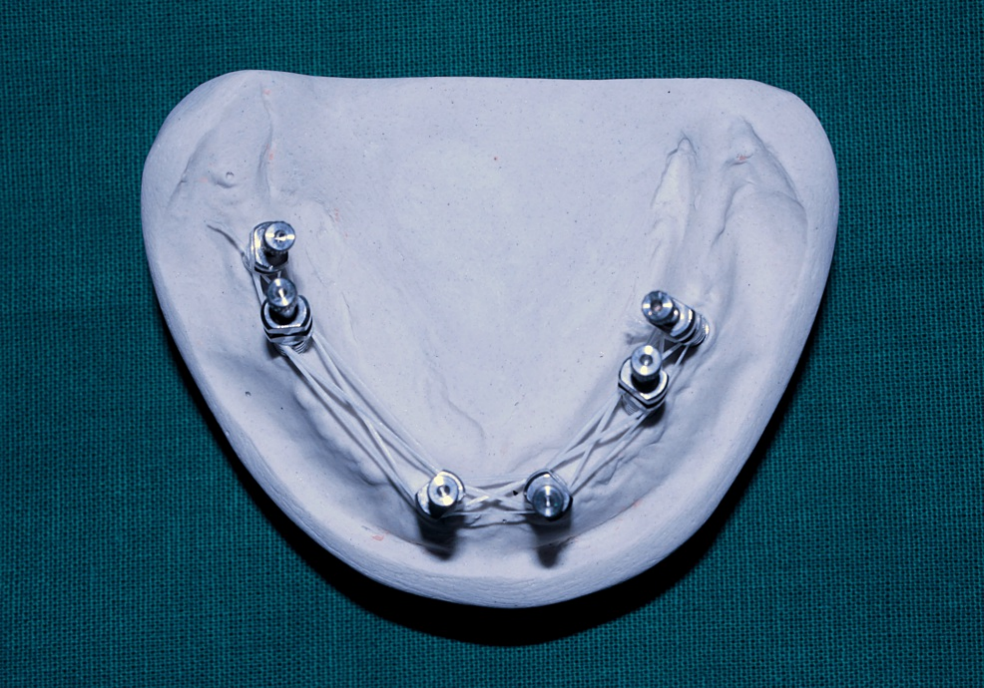
Impression posts splinted using dental floss

**Figure 4:**
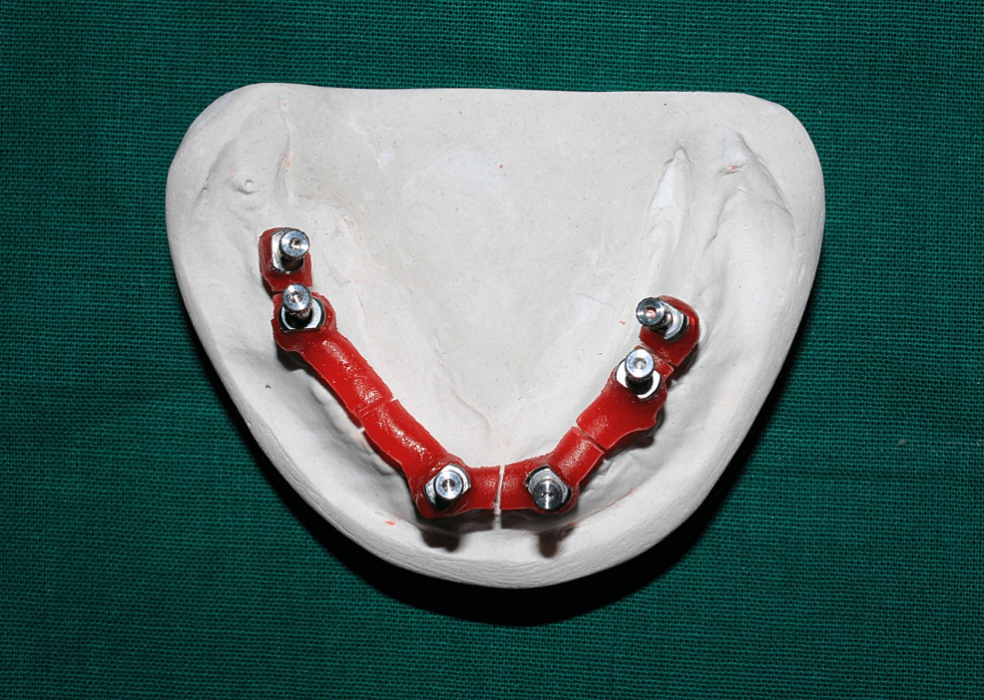
Pattern resin buildup on floss followed by sectioning

The primary cast was poured using type II plaster (Figure 2), which was later used for splinting of impression posts on cast using dental floss and pattern resin (GC Pattern Resin; GC Corp., Tokyo, Japan) (Figure 3), followed by sectioning (Figure 4).

**Figure 5:**
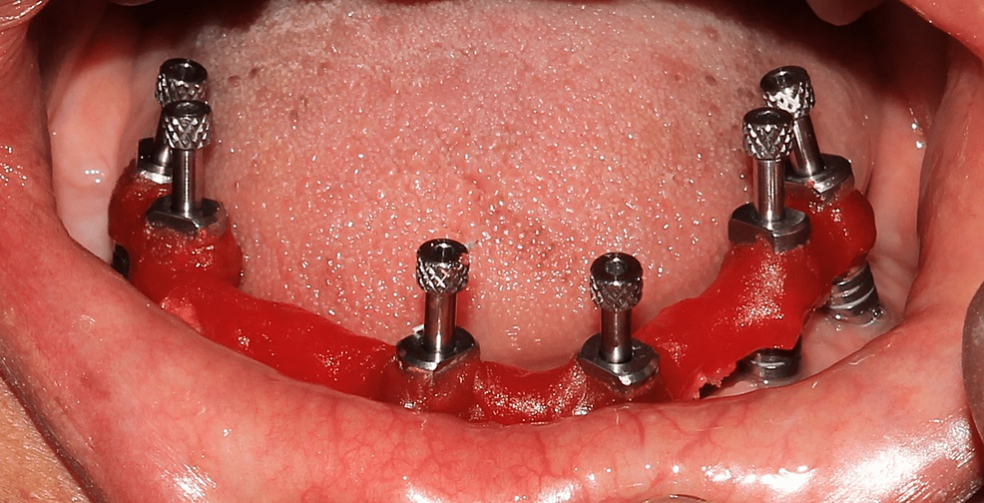
Joining of sectioned segments intraorally using pattern resin after verification of the position of implants

The impression posts were placed intraorally and sectioned segments were joined with pattern resin (Figure 5).

This sectional splinting technique reduced the chairside time and patient discomfort during definitive open tray impression making and decreased polymerization shrinkage. Open tray technique along with sectional splinting increased the accuracy of impression, and hence the precision of passive fit of the implant-supported restorative complex.

**Figure 6:**
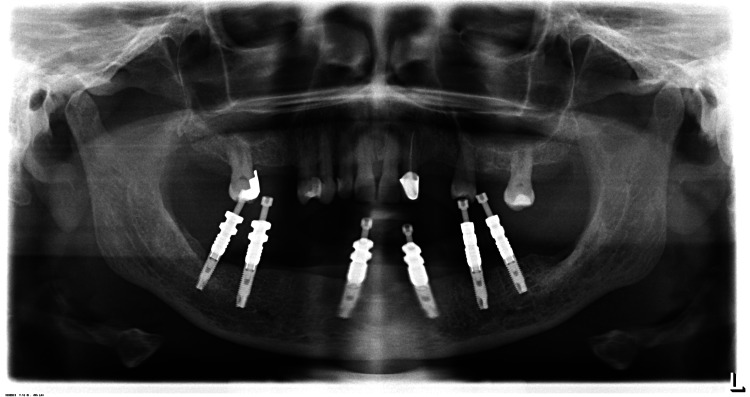
Panoramic radiograph to verify complete seating of impression posts

**Figure 7:**
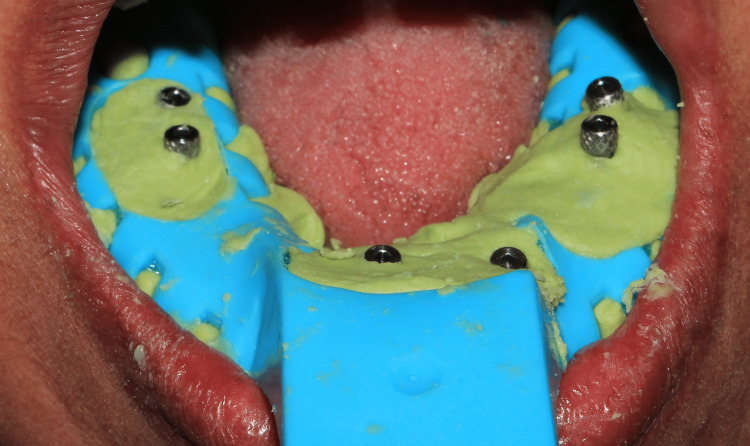
Definitive open tray impression using putty and light body elastomer

A panoramic radiograph was taken to ensure complete seating of impression copings into the implant body (Figure 6), followed by an open tray impression using putty and light body elastomeric (Figure 7).

Implant analogs were attached to the impression copings and a cast was poured in type IV plaster.

**Figure 8:**
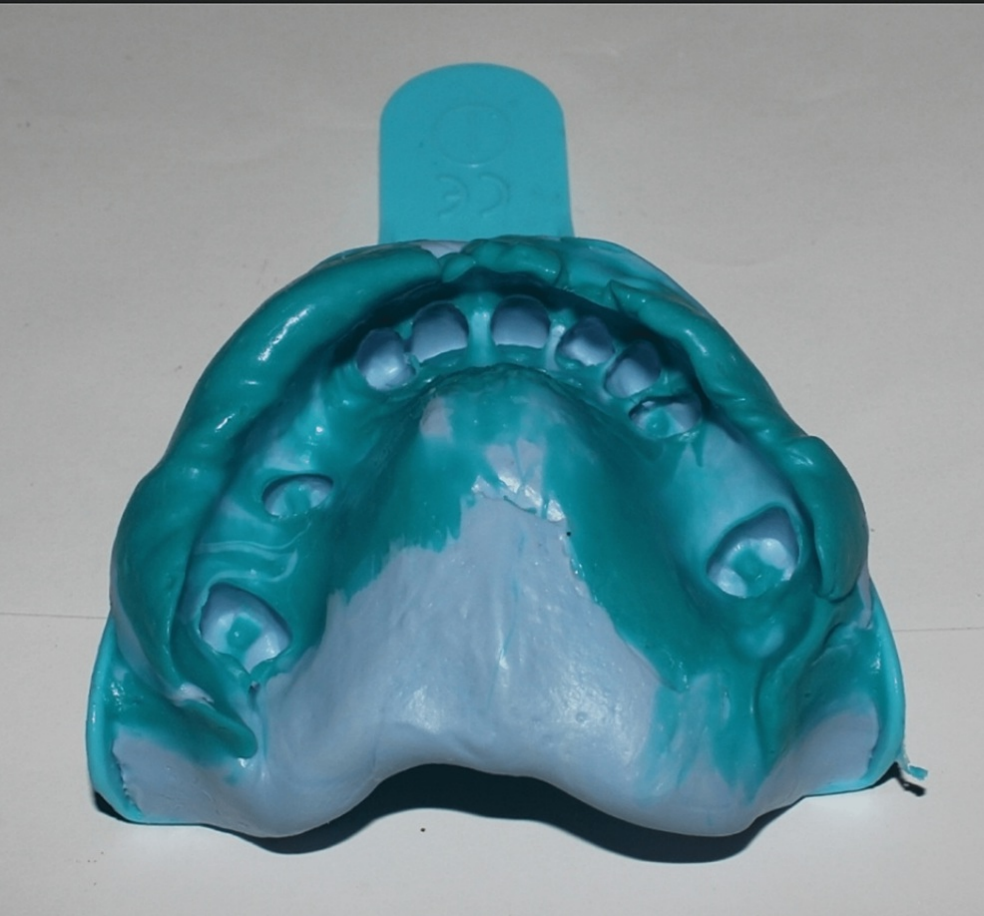
Definitive impression of maxillary arch following rest seat preparation

For the maxillary arch, an anteroposterior strap major connector was planned. Rest seats were prepared for direct retainers on 15, 17, 24, and 27 and indirect retainers on 11, 12, 13, 14, 21, and 22, followed by a definitive impression using putty and light body elastomer (Figure 8).

**Figure 9:**
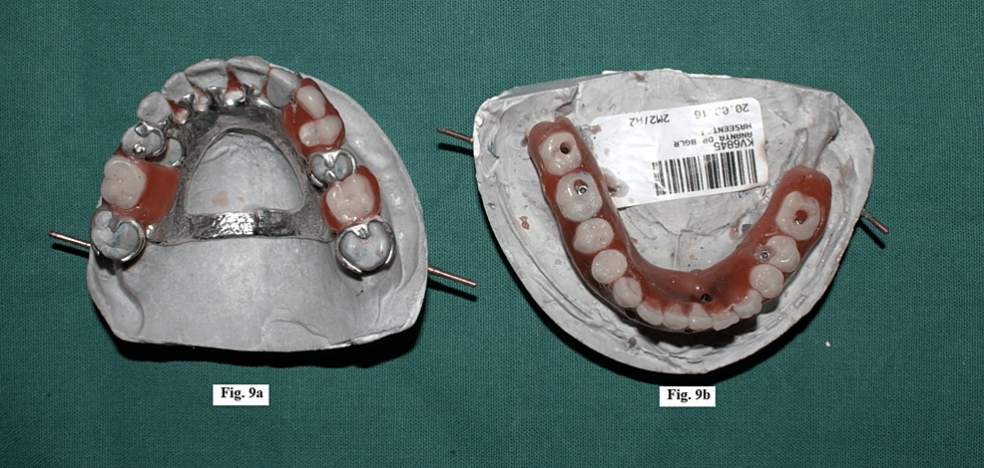
(a) Maxillary cast partial denture framework and teeth arrangement. (b) Mandibular teeth arrangement

**Figure 10:**
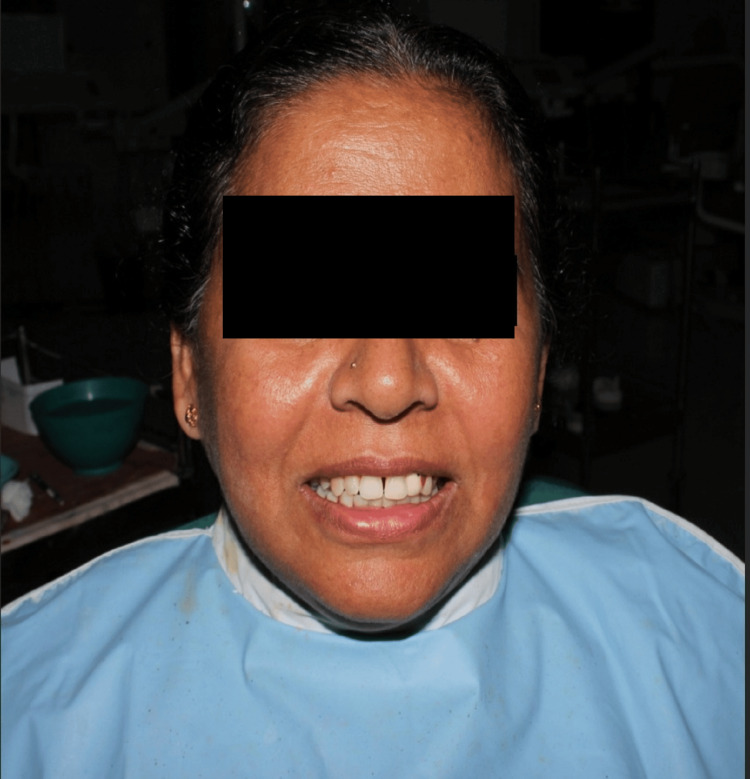
Trial procedure

The cast was poured with type IV plaster. Denture bases and occlusal rims were fabricated and bite registration was done. A face bow transfer was done and the maxillary and mandibular casts were mounted on a semi-adjustable articulator. A maxillary cast partial denture framework was fabricated and maxillary and mandibular teeth arrangement was completed using PeriCeram two-layer ceramic teeth (Figure 9), followed by a trial procedure (Figure 10).

**Figure 11:**
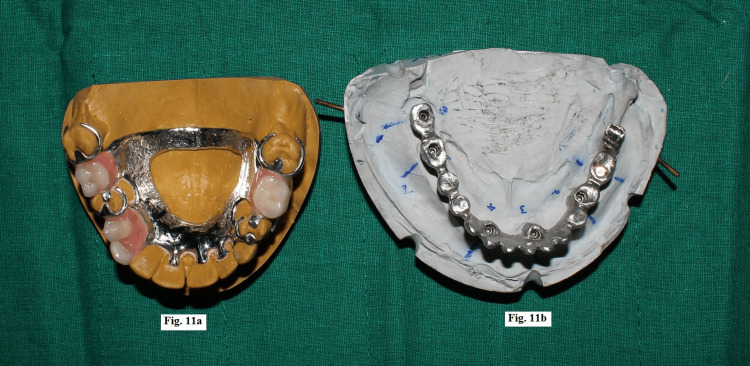
(a) Processed maxillary cast partial denture. (b) Mandibular prosthesis framework

The maxillary cast partial denture was processed and a screw-retained mandibular framework was fabricated, which was later split into two sections using a carborundum disc (Figure 11).

**Figure 12:**
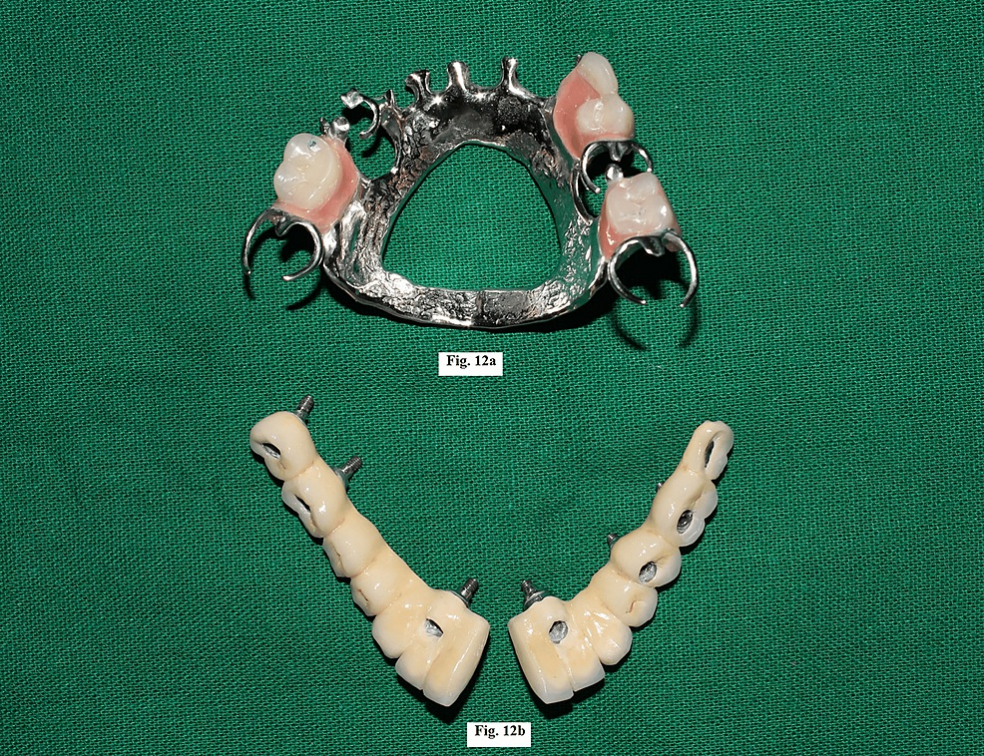
(a) Completed maxillary prosthesis. (b) Splitting of mandibular prosthesis framework and ceramic layering

**Figure 13:**
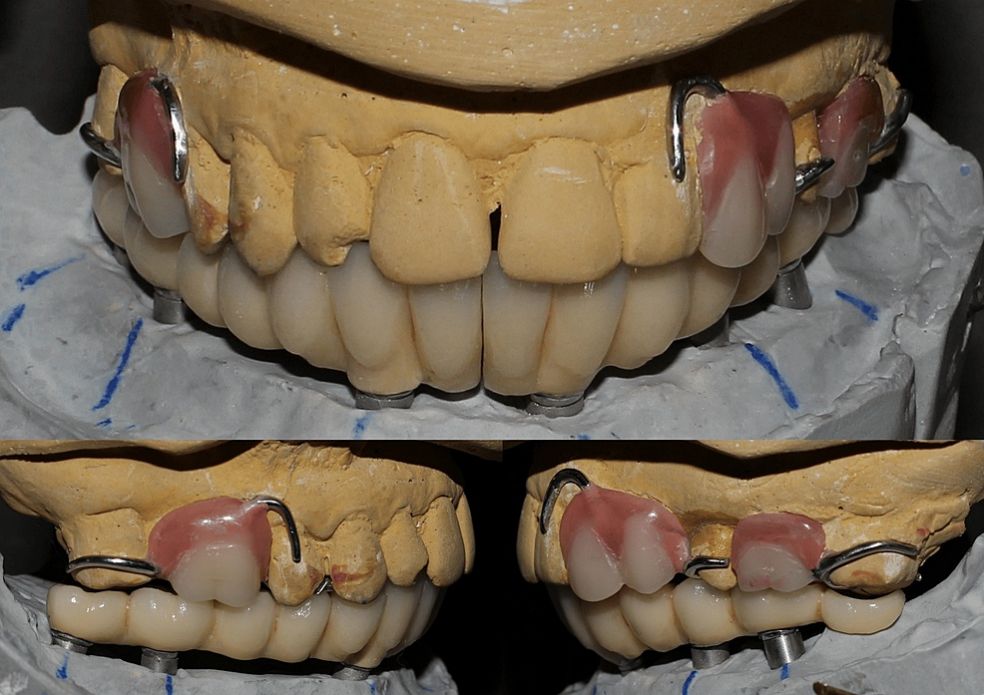
Mounted casts with front, right, and left lateral view of the finished and polished prosthesis in occlusion

**Figure 14:**
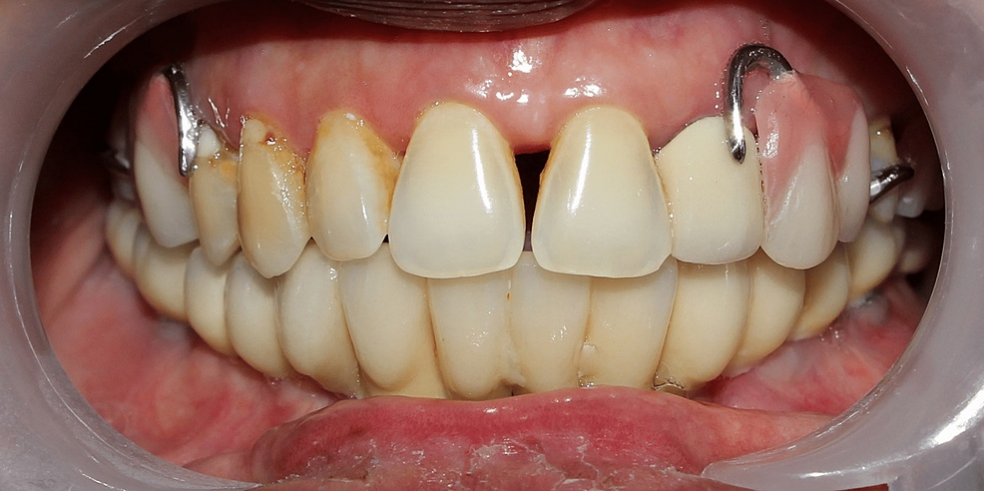
Placement of maxillary and mandibular prosthesis

A trial of the framework was done and it was evaluated for a passive fit. Ceramic layering was done on the framework (Figures 12, 13) and the maxillary and mandibular prostheses were placed (Figure 14), followed by occlusal adjustments.

The separation at the mandibular midline was evaluated using a shim stock to ensure that the sectioned segments of the framework did not come into contact during mandibular opening and protrusive movements. A panoramic radiograph was taken and the patient was educated regarding oral hygiene maintenance and regular recall visits.

The patient was evaluated six months, 12 months, and 18 months after loading and presented no complaints, screw loosening, or significant bone loss.

**Figure 15:**
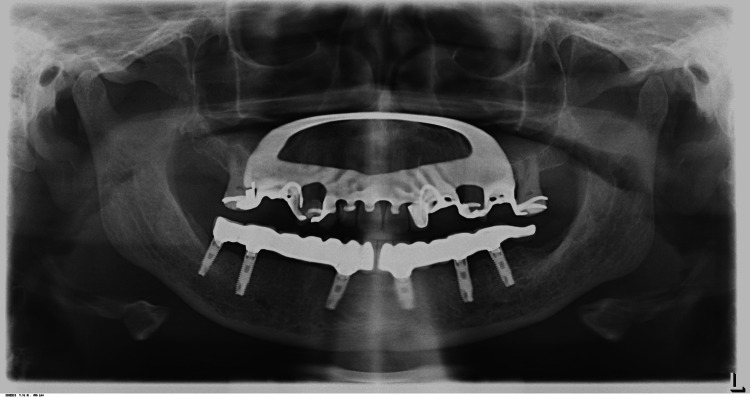
Eighteen-month follow-up panoramic radiograph

## Discussion

The mandibular deformation considering movement or shape is an intricate phenomenon involving an interplay of many muscles of the head and neck region and may even occur without jaw movements.

Based on the work of Hylander [15], symphyseal bending, dorsoventral shear, corporal rotation, and anteroposterior shear were postulated as the four patterns of jaw deformation during mandibular flexure.

In addition to the lateral pterygoid muscle, the superior constrictor muscles, mylohyoid, and platysma also contribute to the condyle's medial movement. During contraction, the depressor muscles of the mandible also produce some changes in the shape of the mandible. During jaw opening and closure, the muscles of the floor of the mouth along with the medial and lateral pterygoid exert a contracting force upon the mandible. During clenching, occlusion, or biting, forces on the mandible and mandibular flexure have been observed [16].

Apart from muscle-related factors, age, gender, muscle strength, bone density, lower gonial angle, and the symphysis height substantially impact the amount of flexure. Patients with considerable mandibular length, smaller gonial angle, and lesser symphyseal area generally show increased mandibular deformation [17].

The flexure of the mandible certainly affects the definiteness of various stages of implant treatment, like osseointegration of the implant, fabrication of implant prosthesis, mastication-related strain distribution within the framework, and crestal bone surrounding implants. Flexural forces incorporate lateral stresses into the implant body resulting in bone loss around the implants, fracture of material, loss of implant fixation, and mouth opening-related discomfort. Hence, it is imperative to consider maxillo-mandibular fixation while planning any implant-supported prosthesis. Median mandibular flexure leads to microdamage near the bone crest and poor osseointegration owing to micromovements around implants [3]. In 1976, Fischman developed splints for the rehabilitation of full arch and evaluated their effect on maxillo-mandibular flexure. He concluded that the flexure of the mandible would be diminished extensively when rigid splinting was carried out. He also found that a short-span splint without rigid attachments will probably lead to better outcomes [10]. However, later it was found that although rigid splinting reduces mandibular flexure, the teeth are not able to flex in their original manner along with the mandible, leading to stress development around them and further bone loss. Thus, mandibular flexure contributes to the alveolar bone destruction. Apart from crestal bone loss, significant buccolingual forces are also generated when fixed and rigid posterior implants are splinted together in a cross-arch restoration. Thus, significant stress is built around distal implants and the superstructure close to the symphysis due to the flexure of the mandible with the symphysis as a fulcrum [11]. Therefore, sectioning of the superstructure at the symphysis level could substantially restore the natural mandibular flexure in function [18]. However, as per a few studies, the necessity of a segmented framework for implant-supported fixed prostheses is currently debated [4,19]. A non-segmented prosthesis could be given if the implant-supported fixed restoration is without a cantilever and if it provides an optimal biomechanical environment with good resistance offsetting the effects of mandibular flexure, especially in cases of the posterior single unilateral framework [4,20].

## Conclusions

Mandibular flexure is a multifactorial phenomenon occurring contemporaneously with jaw movements. It can substantially affect the treatment outcome and prognosis of an implant-supported ceramometal prosthesis. Therefore, it is of utmost importance to take appropriate measures to negate the flexural movement of the jaw during prosthetic rehabilitation. This would help the clinicians deliver a structurally and functionally acceptable prosthesis with an accurate fit and also maintain the health of the surrounding osseous and periodontal tissues. Present studies focus primarily on the completely edentulous mandible. The effect of flexure of the mandible on implant treatment success is uncertain at this stage; however, many studies do suggest that flexure of the mandible should definitely be considered while designing any such prosthesis.
